# Quantifying Metabolic Heterogeneity in Head and Neck Tumors in Real Time: 2-DG Uptake Is Highest in Hypoxic Tumor Regions

**DOI:** 10.1371/journal.pone.0102452

**Published:** 2014-08-15

**Authors:** Erica C. Nakajima, Charles Laymon, Matthew Oborski, Weizhou Hou, Lin Wang, Jennifer R. Grandis, Robert L. Ferris, James M. Mountz, Bennett Van Houten

**Affiliations:** 1 Physician Scientist Training Program, University of Pittsburgh, Pittsburgh, Pennsylvania, United States of America; 2 Howard Hughes Medical Fellow, Howard Hughes Medical Institute, Bethesda, Maryland, United States of America; 3 Department of Pharmacology & Chemical Biology, University of Pittsburgh, Pittsburgh, Pennsylvania, United States of America; 4 Department of Radiology, University of Pittsburgh School of Medicine, Pittsburgh, Pennsylvania, United States of America; 5 Department of Immunology, University of Pittsburgh School of Medicine, Pittsburgh, Pennsylvania, United States of America; 6 Department of Pathology, University of Pittsburgh School of Medicine, Pittsburgh, Pennsylvania, United States of America; 7 Department of Otolaryngology, University of Pittsburgh School of Medicine, Pittsburgh, Pennsylvania, United States of America; Johns Hopkins Medical School, United States of America

## Abstract

**Purpose:**

Intratumoral metabolic heterogeneity may increase the likelihood of treatment failure due to the presence of a subset of resistant tumor cells. Using a head and neck squamous cell carcinoma (HNSCC) xenograft model and a real-time fluorescence imaging approach, we tested the hypothesis that tumors are metabolically heterogeneous, and that tumor hypoxia alters patterns of glucose uptake within the tumor.

**Experimental Design:**

Cal33 cells were grown as xenograft tumors (n = 16) in nude mice after identification of this cell line's metabolic response to hypoxia. Tumor uptake of fluorescent markers identifying hypoxia, glucose import, or vascularity was imaged simultaneously using fluorescent molecular tomography. The variability of intratumoral 2-deoxyglucose (IR800-2-DG) concentration was used to assess tumor metabolic heterogeneity, which was further investigated using immunohistochemistry for expression of key metabolic enzymes. HNSCC tumors in patients were assessed for intratumoral variability of ^18^F-fluorodeoxyglucose (^18^F-FDG) uptake in clinical PET scans.

**Results:**

IR800-2-DG uptake in hypoxic regions of Cal33 tumors was 2.04 times higher compared to the whole tumor (p = 0.0001). IR800-2-DG uptake in tumors containing hypoxic regions was more heterogeneous as compared to tumors lacking a hypoxic signal. Immunohistochemistry staining for HIF-1α, carbonic anhydrase 9, and ATP synthase subunit 5β confirmed xenograft metabolic heterogeneity. We detected heterogeneous ^18^F-FDG uptake within patient HNSCC tumors, and the degree of heterogeneity varied amongst tumors.

**Conclusion:**

Hypoxia is associated with increased intratumoral metabolic heterogeneity. ^18^F-FDG PET scans may be used to stratify patients according to the metabolic heterogeneity within their tumors, which could be an indicator of prognosis.

## Introduction

Intratumoral heterogeneity is gaining attention as a contributor to tumor recurrence and incomplete response to therapy [1]–[4]. Fluctuating oxygenation in tumors is well documented [5]–[13] and oxygen availability is a major determinant of whether glycolytic metabolism or the more efficient oxidative pathways are used for ATP production [14]–[18]. A tumor's response to variable oxygen levels can lead to metabolic flexibility and type of intratumoral symbiosis, in which lactate produced by hypoxic, glycolytic cells provides a fuel source for oxygenated cells performing oxidative phosphorylation (OXPHOS) [7], [17], [19], [20]. While the elevated glycolytic activity of malignant cells has long dominated the study of cancer metabolism [1], [3], [21], increasing evidence demonstrates tumors can consume a variety of metabolites such as lactate, glutamine, and fatty acids [5], [7], [9], [11], [13], [22]. We refer to the ability of cancer cells to alter their nutrient consumption in response to shifting oxygenation as metabolic flexibility. We believe metabolically flexible malignant cells are more likely to form aggressive tumors due to their ability to adapt to environmental pressures.

Tumor hypoxia is a known predictor of worsened prognosis in head and neck squamous cell carcinoma (HNSCC) [14], [16], [18], [23], [24]. We believe this outcome is partially due to the metabolic heterogeneity that results from differential oxygenation within a tumor. To our knowledge, a thorough assessment of intratumoral metabolic heterogeneity in HNSCC remains to be performed. While markers of metabolic heterogeneity have been identified in histologic examinations of HNSCC tumors [19], [20], [25], no studies have mapped metabolic diversity in real-time and *in vivo*, using high resolution fluorescent molecular tomography (FMT). We sought to determine the role of hypoxia in driving metabolic changes in HNSCC tumors.

To validate the metabolic flexibility of HNSCC, we confirmed that hypoxic conditions increased glucose consumption and lactate production in two HNSCC cell lines *in vitro*. The cell line (Cal33) with greater metabolic flexibility was use to grow xenograft tumors. Real-time, *in vivo* fluorescent molecular tomography was performed upon the tumors to measure tumor hypoxia, vasculature, and heterogeneity of glucose uptake. Finally, we extended our findings of xenograft tumor heterogeneity to clinical practice by measuring metabolic heterogeneity in ^18^F-fluorodeoxyglucose (FDG) PET-CT scans of four HNSCC tumor patients.

## Methods

### Tissue Culturing

Cal33, an epidermoid head and neck squamous cell carcinoma cell line derived from an oral squamous cell carcinoma [21], [26], was kindly provided by Dr. J.L. Fischel (Centre Antoine Lacassagne, Nice, France). Cell line OSC19 was derived from a squamous cell carcinoma of the tongue [1], [22]. Both cell lines were validated using short tandem repeat in April 2011 expanded and frozen down. Cell pellets were kept frozen in liquid nitrogen and newly thawed cells were used from May 2012 to May 2013. Cells were maintained in DMEM containing 25 mM glucose (Gibco) with 10% fetal bovine serum and 1% penicillin-streptomycin at 37C in a humidified incubator with 5% CO_2_, 95% air. Hypoxic culturing was performed in 2% O_2_, 7.5% CO_2_.

### Measuring metabolite consumption and production

Cells were incubated in 21% or 2% O_2_ for 12, 24, and 48 hours in culture media described above. At each time point, a media aliquot was harvested and the cell count in each dish was recorded after trypsinization. Concentrations of glucose and lactate in the original and culture media were measured using an Accutrend Plus metabolite meter (Roche) [5], [23], [24].

### Immunoblotting

Cells were lysed on ice immediately after removal from an incubator with TNG cell lysis buffer (Cell Signaling) containing complete Mini protease inhibitor (Roche). Proteins were separated by electrophoresis on Novex 4–12% Bis-Tris gels (Invitrogen), and transferred to nitrocellulose membrane (Bio-Rad). Membranes were blocked in 10% milk in PBS with Tween 20, except when being probed for HIF-1α, in which case 5% milk TBS solution with Tween 20 was used. The following antibodies were used to probe the membranes: hypoxia inducible factor-1α (HIF-1α) (Epitomics, 1∶500), lactate dehydrogenase subunit M (LHD-M) (Santa Cruz Biotechnology, 1∶1000), pyruvate dehydrogenase kinase 1 (PDHK1) (Cell Signaling, 1∶1000), ATP synthase subunit β (ATP5β) (Abcam, 1∶1000), carbonic anhydrase nine (CAIX) (Abcam, 1∶1000), and β-actin (Sigma Aldrich, 1∶30,000). Densitometry was performed using ImageJ.

### Evaluation of cellular rates of glycolysis and oxidative phosphorylation

The oxygen consumption rate and extracellular acidification rate were recorded in real-time using a Seahorse XF24 Extracellular Flux Analyzer (Seahorse Biosciences) using a slight modification of a previously published protocol [15], [25]. See Methods S1 for further method details.

### Nude mouse xenograft model

5×10^5^ Cal33 cells were injected subcutaneously into the left flank of 6-week-old female Foxn1 nude mice (Harlan Laboratories) in two sets of experiments with ten mice each. During the first experiment, mice also received an injection of 2.5×10^5^ cells into the right flank. During the second experiment, the mice received 5×10^5^ Cal33 cells into the left flank only. Tumors were palpable after 10 days, and tumors were measured every other day using calipers. The University of Pittsburgh Institutional Animal Care and Use Committee (Pittsburgh, PA) approved all the animal experiments (IACUC protocol: 12070675).

### Fluorescent molecular tomography (FMT) and image analysis

Mice were injected intravenously (IV) with 2 nmol of HypoxiSense 680 (PerkinElmer) 48 hours prior to imaging, (Table SI). Mice were injected IV 24 hours later with 2 nmol AngioSense 750 (PerkinElmer) and 10 nmol IRDye-800CW-2-deoxyglucose (IR800-2-DG) (Li-COR Biosciences) (Table SI). 24 hours after the last IV injection, mice were anesthetized with isofluorane, placed into a biplanar imaging cassette, and were imaged in three-dimensions using a VisEn FMT2500 Quantitative Tomography In Vivo Imaging System (PerkinElmer) [26], [27]. Slices were acquired with a thickness of 0.25 mm in z-direction. Two dimensional images were created from the FMT DICOM files using ITK-SNAP software [1], [6], [10]. Each plane represents a 250 µm thick slice of a tumor. See Materials S2 for further method details.

### Anatomical tumor measurement

To validate that the FMT-based ROI was appropriately placed, one mouse was sacrificed immediately after FMT imaging while remaining in the FMT cassette to maintain body position. Using a specialized adapter to hold the FMT cassette, the body underwent MRI scanning with a 7Tesla CliniScan (Bruker Corp.). 3D tumor reconstruction and volume measurement was performed using manually drawn, serial ROIs in ITK-SNAP software.

### Immunohistochemistry

Xenograft tumors were excised and flash frozen in OTC (Sakura). One tumor was sectioned while frozen in OCT. The remaining tumors were fixed in formalin, and embedded in paraffin. Before sectioning, a 25-gauge needle was inserted into the tumors to serve as a fiduciary marker. 5 µm tissue slices were stained for ATP5β (1∶50), CAIX (1∶500), HIF-1α (1∶100), and LDH-M (1∶250) using the same antibodies as those used for western blotting. Slices were counterstained with hematoxylin. The following positive controls were used, ATP5β (normal murine kidney), HIF-1α (normal kidney), LDH-M (normal colon), and CAIX (renal cell carcinoma). A certified pathologist approved the quality of the staining. Slides were scanned at 20× on Aperio Imagescope software. Images of whole tumor sections were captured at 20× or 40×. Images of regions within tumor slices were captured at 200×.

A certified pathologist, who was blinded to the FMT imaging results, selected 20 representative regions in two tumors that had shown a strong hypoxic signal and two that had not. The staining intensity was measured by positive pixel count (Aperio version 9). The protein expression was quantified in each region by multiplying the staining intensity by the positive area percentage.

### Patients

Four patients with HNSCC tumors gave written consent to be included in this study. None of the patients had received any therapy for their malignancies prior to the baseline positron emission tomography-computed tomography (PET-CT) scan. This protocol was approved by the University of Pittsburgh Institutional Review Board (IRB number PRO09080141).

### FDG-PET/CT imaging

PET scans were performed using DST16 PET-CT (GE) that has a spatial resolution of 0.5 cm. Patients fasted for at least four hours prior to receiving 15 mCi ^18^F-FDG intravenously. For this study, data acquired over the 5-minute interval from 63 to 68 minutes post injection were used in the analysis. A low-dose CT scan without contrast was also acquired for attenuation and scatter correction.

Data were reconstructed iteratively (2 iterations of 28 subsets) following Fourier rebining (FORE) into a 128×128×47 (axial) matrix with voxel size 4.7 mm×4.7 mm×3.3 mm. Reconstruction was accomplished using the manufacturer's software and included corrections for attenuation, scatter, random coincidences, and deadtime.

### Heterogeneity analysis of PET images

Tumor segmentation was standardized across patients using the PETEdge algorithm of the MIMvista software package (MIM Software) [5], [17]. PETEdge delineates the tumor border in the presence of background signal by assessing changes in ^18^F-FDG spatial gradients. The PETEdge-drawn ROI was designated as ROI-E.

Quantification in PET is complicated by the modality's limited spatial resolution that results in a blurring of voxel intensities [15], [28], a serious challenge for an analysis of heterogeneity. An additional problem is the mixing of background signal into image voxels within the tumor, resulting in an artifactual decrease in apparent tumor signal intensity at the tumor boundary. For the PET scanner used in this study, the spatial resolution is on the order of 0.6–0.7 cm full-width at half-maximum [27], [29]. This issue was addressed by contracting the boundary of ROI-E by 0.5 cm circumferentially. The new, contracted ROI is labeled ROI-C. For example, if ROI-E were a sphere of *radius* 5 cm, than ROI-C would be a sphere of radius 4.5 cm.

The mean ^18^F-FDG concentration and standard deviation across voxels were calculated within ROI-C. The dimensionless coefficient of variation (CV), defined as the standard deviation divided by the mean, was computed as a measure of heterogeneity.

## Results

### Hypoxia exposure alters Cal33 cellular metabolism, decreasing OXPHOS and increasing glycolysis

**Figure 1:**
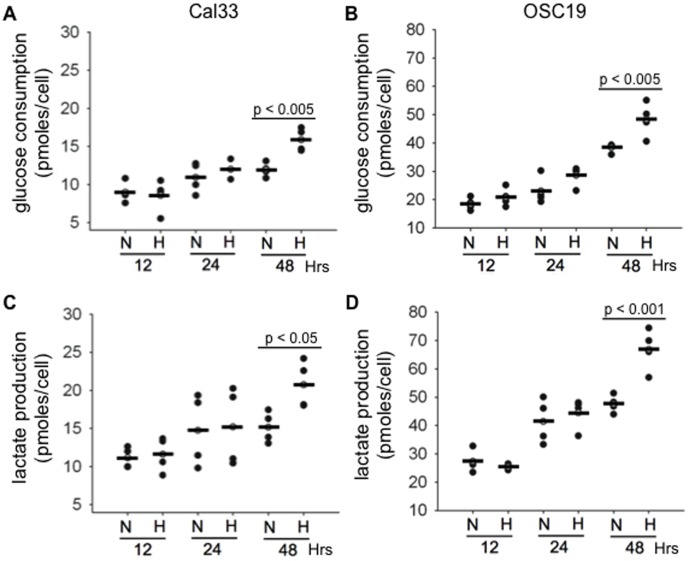
Glucose consumption and lactate production increase under hypoxic conditions. A and B, Cal33 and OSC19 cells consume more glucose after 48 hours of hypoxic culturing than cells grown in normoxic conditions. C and D, a concomitant rise in lactate production was detected in media from Cal33 and OSC19 cells grown in hypoxic conditions for 48 hours. OSC19 cells are more glycolytic than Cal33 cells. Each dot represents one of four replicate dishes used for each time point and culturing condition. The experiment was performed twice with two replicate dishes. P values indicate a significant difference in metabolite concentrations and were calculated using a two-way ANOVA.

It was imperative to detect HNSCC metabolic flexibility in response to hypoxia *in vitro* prior to the investigation of tumor metabolic heterogeneity *in vivo*. Cal33 and OSC19 cells were selected for an initial investigation because these lines establish xenografts successfully [6], [10], [30]. Cal33 and OSC19 cells increased their glucose consumption by 38% (p<0.005) and 26% (p<0.005) respectively, when cultured in 2% O_2_ for 48 hours as compared to cultures grown in 21% O_2_ (Fig. 1A and B). Both cell lines had a corresponding increase in lactate production after 48 hours of hypoxia exposure (Cal33 37%, p<0.05; OSC19 40%, p<0.001) (Figs. 1C and D). The difference in glucose consumption or lactate production was insignificant at earlier time points of 2% or 21% O_2_ culturing. These measurements also demonstrated that OSC19 cells were approximately two and a half times more glycolytic as compared to Cal33 cells in normoxic and hypoxic culturing conditions.

**Figure 2:**
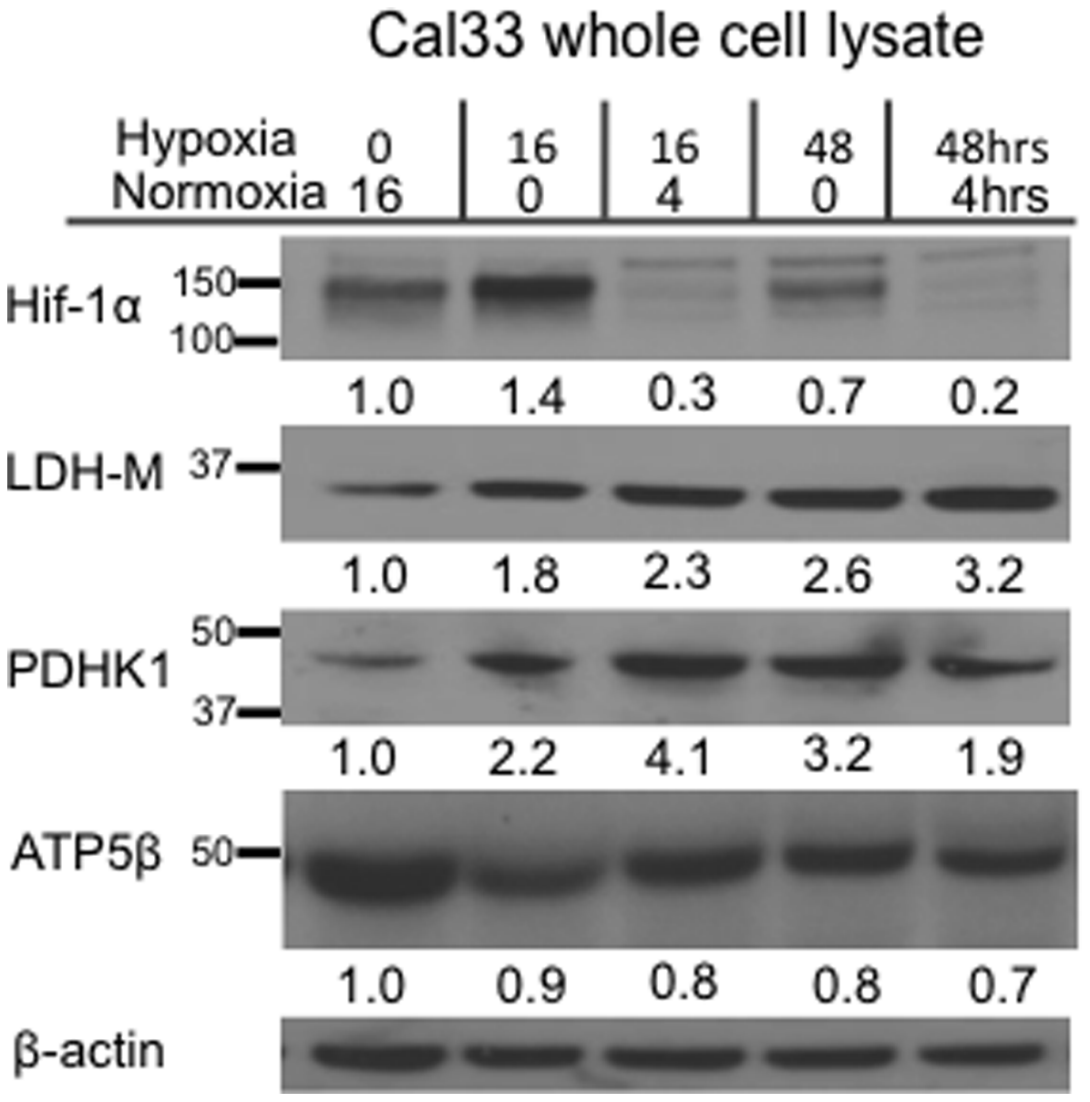
Cal33 exposure to hypoxia leads to increased expression of HIF-1α, LDH-M, and PDHK1. Western blot analysis of changes in HIF-1α, LDH-M, and PDHK1 expression in whole cell lysate from Cal33 cultures grown in normoxic (21%), hypoxic (2%), or hypoxic (2%) followed by normoxic (21%) conditions. Cells cultured in 21% oxygen for 16 hours serve as the control for basal protein expression levels. Densitometry values were calculated using ImageJ. Images are representative of duplicate blots.

As Cal33 cells showed the greatest increase in glucose consumption after hypoxia exposure, this cell line was selected to assess for altered expression of proteins involved in the metabolic response to hypoxia. HIF-1α is a transcription factor that regulates acute cellular response to hypoxia [7], [9], [11], [13], [15], [17]. HIF-1α was stabilized in Cal33 cells after 16 hours of hypoxia, but was degraded rapidly when cells were maintained in normoxic conditions for four hours after hypoxic incubation (Fig. 2). Expression of HIF-1α-regulated enzymes lactate dehydrogenase subunit M (LDH-M), and pyruvate dehydrogenase kinase 1 (PDHK1) remained significantly elevated at 16 and 48 hours of hypoxia exposure and after normoxia exposure. These enzymes promote anaerobic glycolysis by shunting carbons away from the OXPHOS pathway. These data support our previous findings of higher glycolytic rate and increased lactate production in Cal33 cells cultured in hypoxia for 48 hours (Fig. 1A and C). ATP5β expression served as an indicator of mitochondrial ATP synthase levels, as the synthase complex is involved in OXPHOS pathway. ATP5β expression decreased by approximately two-fold in Cal33 cells exposed to prolonged hypoxia, suggesting that mitochondrial ATP synthase levels drop in response to limited oxygenation.

### Cal33 cells reduce OXPHOS activity in response to acute hypoxia

**Figure 3:**
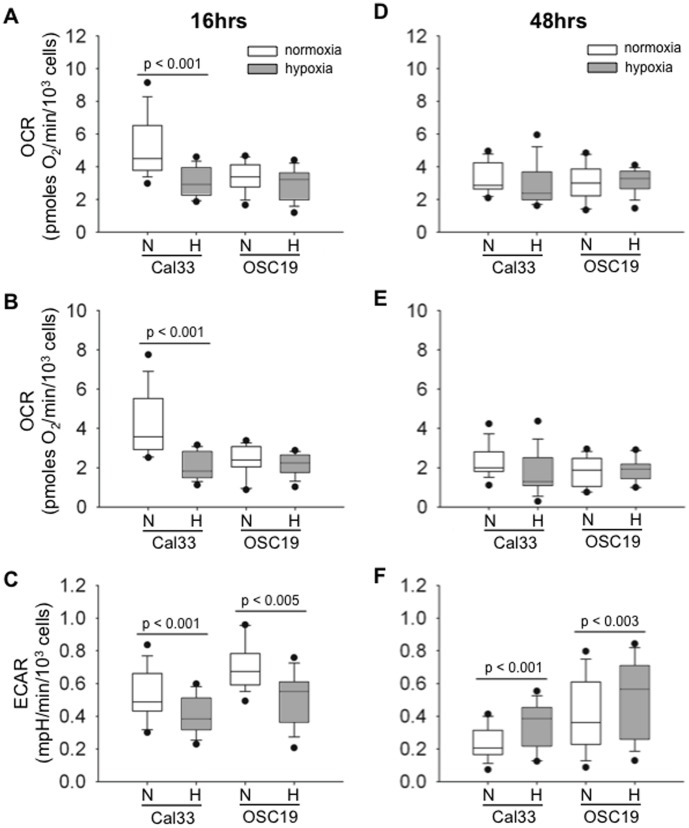
Cal33 cells demonstrate greater metabolic flexibility in response to hypoxia than OSC19 cells. After 16 or 48 hours in 2% (hypoxia) or 21% oxygen (normoxia), 4×10^4^ Cal33 or OSC19 cells were plated in a Seahorse XF24 Extracellular Flux Analyzer plate. The Seahorse Analyzer records rates of OXPHOS & glycolysis via the oxygen consumption rate (OCR) and the extracellular acidification rate (ECAR) of each sample culture. A, basal OCR recorded at the assay start after 16 hour incubation. B, ATP-linked OCR is the difference between the basal OCR and the OCR after the addition of oligomycin to sample cultures (see Figure S1). C, Basal ECAR recorded at the assay start. Three experiments were performed at each time point, with five samples per condition. Boxes represent the interquartile range, horizontal lines indicate the median, the T-bars indicate the range, and individual points are outliers. P values were determined by two-way ANOVA. N = normoxia (21% oxygen), H = hypoxia (2% oxygen). D, Basal OCR recorded at assay start after 48 hour incubation. E, ATP-linked OCR recorded for 48 hour cultures. F, Basal ECAR recorded after 48 hour incubation.

Having identified metabolic flexibility and hypoxia-induced increased glycolysis in Cal33 cells, we sought to further validate the glycolytic changes and to determine whether acute hypoxia altered rates of OXPHOS in this cell line. The Seahorse Flux Analyzer assay measures cellular glycolytic and OXPHOS rates simultaneously. The glycolytic rate is measured via changes in the extra-cellular acidification rate (ECAR) of a cell culture. OXPHOS activity is assessed via the oxygen consumption rate (OCR) (Fig. S1). After 16 hour incubation, Cal33 cells cultured in hypoxic conditions demonstrated a dramatic decrease (41%) in their basal OCR as compared to cells cultured in 21% O_2_ (Fig. 3A).

The addition of pharmacological inhibitors enables measurements of OXPHOS associated with ATP production (ATP-linked OCR, described in Fig. S1). After 16 hours of hypoxia exposure, Cal33 ATP-linked OCR was reduced by 53% as compared to normoxic treated controls (Fig. 3B). OSC19, the more glycolytic of the cell lines (Fig. 1B), did not demonstrate significant alterations in OXPHOS activity when exposed to hypoxic conditions (Fig. 3A and B). Surprisingly, Cal33 and OSC19 had reduced glycolytic activity after 16 hours of hypoxic culturing (24 and 27% respectively) (Fig. 3C), suggesting that these cells suppressed their glycolytic metabolism initially under reduced oxygen conditions. This would help to explain why we did not detect a significant difference in glucose consumption at earlier time points as shown in Fig. 1.

To confirm the increase in glucose consumption seen at 48 hours in Figure 1, the Seahorse assay was also performed on Cal33 and OSC19 cultures grown in normoxic or hypoxic conditions for 48 hours. Both Cal33 and OSC19 cells revealed increased glycolytic activity (40% and 30% respectively) after 48 hours of hypoxic exposure as compared to cells exposed to normoxic conditions. (Fig. 3F) Interestingly, there was no significant difference between basal and ATP-linked OCR values for hypoxic and normoxic cultures of both cell lines after 48 hrs of continuous growth under these conditions.

The Seahorse data substantiated the finding that Cal33 are more metabolically flexible than OSC19 cells, displaying lower initial levels of glycolysis and a significant decrease in OXPHOS after hypoxia exposure. As a result, Cal33 was selected as the xenograft model for our *in vivo* investigation of intratumoral metabolic heterogeneity.

### Hypoxic tumor regions have increased 2-DG uptake

**Figure 4:**
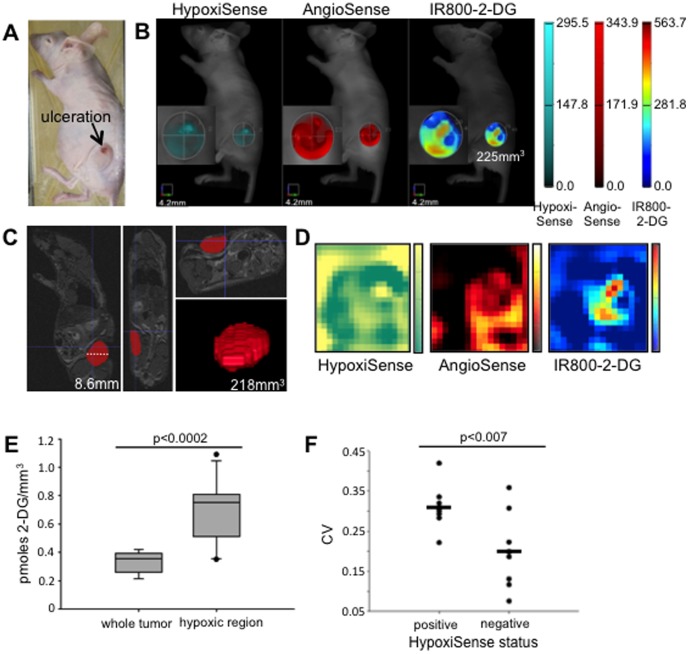
2-DG uptake in Cal33 xenografts is heterogeneous and is associated with HypoxiSense accumulation. A, Nude mouse with an ulcerated Cal33 xenograft tumor. B, 3D reconstructions of FMT scans capturing HypoxiSense, AngioSense, and IR800-2-DG signal within the xenograft tumor as shown in A. A manually placed ROI measures the tumor volume. C, Anatomic tumor measurement was calculated from MRI slices. Sagital, axial, and coronal MRI slices of the mouse shown in A and B, with 3D tumor reconstruction. D, two dimensional reconstructions of FMT scans at 2 mm depth within the tumor. E, box plots comparing IR800-2-DG concentration in whole tumors and HypoxiSense-concentrated regions within them (n = 8). Boxes represent the interquartile range, the horizontal line indicates the median, the T-bars indicate the range, and individual points are outliers (p, student t-test). F, Tumors were grouped by the presence or absence of HypoxiSense signal within the tumor (n = 9 for HypoxiSense positive, n = 7 for HypoxiSense negative). The coefficient of variance (CV) for IR800-22-DG concentration within the tumor was calculated (p value was generated using a student's t-test). Each point represents a tumor. Bars indicate the average CV value for the HypoxiSense positive or negative group.

We next investigated whether discrete regions of Cal33 xenograft tumors had variable glucose uptake, and whether areas of increased glucose uptake would correlate to hypoxic tumor regions. After xenograft Cal33 tumors had grown for approximately two weeks, xenograft-bearing mice were injected with HypoxiSense [26], [28], AngioSense [17], [29], [31], and IR800-2-DG [8], [12], [30], [32], markers of hypoxic tissue, tumor vasculature, and glucose uptake respectively (Table S1). Serial FMT scans recorded intratumoral dye concentration (pmoles/mm^3^). One tumor had a necrotic ulceration on its surface (Fig. 4A), and this same region contained the strongest HypoxiSense signal in the tumor (Fig. 4B). One mouse underwent whole body MRI scanning immediately after FMT imaging to ensure that the FMT-based tumor size measurements were accurate. The tumor was reconstructed manually in 3D from the MRI slices (Fig. 4C). The FMT-based and MRI-based measurement of the tumor resulted in similar tumor volumes (225 mm^3^ and 218 mm^3^, respectively).

Significant heterogeneity in HypoxiSense, AngioSense, and IR800-2-DG tumor uptake was visible from 2D and 3D tumor images (Fig. 4B, D). HypoxiSense was detectable at a set threshold in nine of the 16 tumors imaged, usually within a small focused region of the tumor (Fig. 4B, and Fig. S3). For tumors containing an area of HypoxiSense accumulation, we assessed whether Hypoxisense-positive tumor areas coincided with areas of elevated IR800-2-DG. 3D ROIs were placed around the whole tumor and HypoxiSense-concentrated tumor regions. Three tumors had multiple areas of HypoxiSense accumulation. The IR800-2-DG concentration of the entire eight tumors was compared to the IR800-2-DG concentration in 12 hypoxic regions within those same tumors. The increase in HypoxiSense concentration between whole tumor and hypoxic region ROIs ranged between 2.5 to 225 fold. To compare IR800-2-DG uptake in the whole tumor and HypoxiSense-concentrated regions, these ROIs were then applied to the identical tumor location on the FMT scan of IR800-2-DG. The IR800-2-DG concentration within hypoxic tumor regions was on average 2.04-fold greater than the IR800-2-DG concentration in the whole tumor (Fig. 4E).

We were also interested in whether the degree of IR800-2-DG heterogeneity varied between tumors in which HypoxiSense was detectable and those tumors in which it was undetectable. To achieve this aim, we calculated the coefficient of variation (CV) of IR800-2-DG signal within each tumor by dividing the standard deviation of IR800-2-DG signal by the mean IR800-2-DG signal within each whole-tumor ROI. Intriguingly, those tumors (n = 9) that were strongly positive for HypoxiSense accumulation had an average IR800-2-DG CV value (0.31) that was approximately 33% greater than the CV value (0.2) for tumors in which HypoxiSense was undetectable at the designated threshold (n = 7) (Fig. 4F).

### Expression of metabolic proteins associated with glycolysis is heterogeneous in Cal33 xenograft tumors

**Figure 5:**
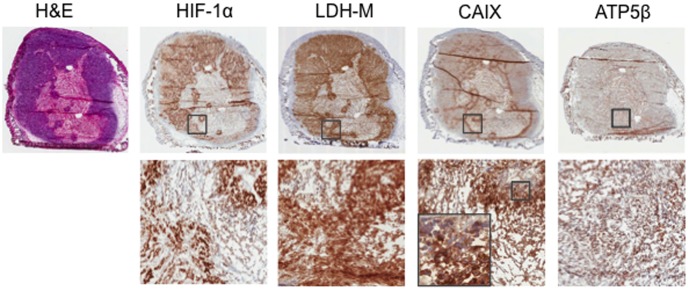
Heterogeneous staining pattern of HIF-1α, LDH-M, and CAIX expression in xenograft tumors reflects presence of hypoxia. Representative images of HypoxiSense Cal33 tumor sections were probed for HIF-1α, LDH-M, and CAIX expression by immunohistochemistry (IHC) as described in materials and methods. Gray boxes indicate the region of the tumor shown at 10× magnification. Slides were scanned at 20× on Aperio Imagescope software. Images of whole tumor sections were captured at 20×. Images of regions within tumor slices were captured at 200×. Additional inset of CAIX shows cell-surface staining of this protein in peri-necrotic areas.

To confirm our *in vivo* findings of regional hypoxia and metabolic heterogeneity in the xenograft tumors, immunohistochemistry was performed on four tumors to detect HIF-1α, LDH-M, ATP5β, and CAIX expression (Fig. 5 and Figure S4). Immunohistochemistry data are presented for one of two tumors that were HypoxiSense positive on FMT. The additional two tumors that were assessed by IHC were HypoxiSense negative (data not shown).

Hematoxylin and eosin staining revealed necrotic tumor centers. HIF-1α expression was strongest along the necrotic tissue border, suggesting that the tissue breakdown was associated with poor oxygenation. CAIX expression followed a similar pattern, however, the location of the protein expression was additionally informative. CAIX was found at the cell surface of viable tumor cells adjacent to the necrotic areas, while in portions of viable tissue more distant from the necrotic area, CAIX was confined to the cytoplasm. This distinction in CAIX localization explains why CAIX levels appeared unchanged on immunoblotting when Cal33 cells were exposed to 2% oxygen (Fig. S2), as this technique could not discern the location of protein expression. LDH-M expression followed a pattern similar to HIF-1α, and was strongest in regions of viable tissue surrounded by necrosis. ATP5β expression was strongest in regions of viable tissue. These patterns of expression were identifiable in the HypoxiSense positive and negative tumors.

To assess the heterogeneity of protein expression in the tumors, 20 representative regions of each tumor section for each of the four tumors, and were scored for positive pixel count. The mean, standard deviation (SD), and CV were calculated from the 20 scores (Table 1). CV values for the expression of HIF-1α, CAIX, and ATP5β were higher in the HypoxiSense positive tumors, indicating greater heterogeneity of expression in tumors deemed hypoxic by FMT scan. The CV values of LDH-M expression were similar for HypoxiSense positive and negative tumors.

**Table 1:** HIF-1α, ATP5β and CAIX expression have greater heterogeneity in HypoxiSense-positive tumors as compared to HypoxiSense-negative tumors.

Hypoxi-Sense status	HIF-1α			LDH-M			CAIX			ATP5β		
	Mean	SD	CV	Mean	SD	CV	Mean	SD	CV	Mean	SD	CV
Positive	123.72	27.78	**0.22**	134.17	10.75	**0.08**	97.40	31.83	**0.33**	66.26	12.60	**0.19**
Positive	69.23	15.78	**0.23**	148.74	8.73	**0.06**	138.65	43.32	**0.31**	51.20	11.42	**0.22**
Negative	79.30	11.60	**0.15**	151.01	7.38	**0.05**	170.69	14.65	**0.09**	79.9	6.07	**0.08**
Negative	90.73	17.37	**0.19**	156.80	6.89	**0.04**	151.01	7.38	**0.05**	85.02	10.06	**0.12**

Mean, standard deviation (SD), and coefficient of variance (CV) of staining were quantified by measuring positive pixel count in 20 representative regions of each tumor slice using Aperio version 9. Quantification of protein expression in each region was calculated by multiplying the staining intensity by the positive area percentage as described in materials and methods.

### Measuring intratumoral metabolic heterogeneity in ^18^F-FDG PET scans

**Figure 6:**
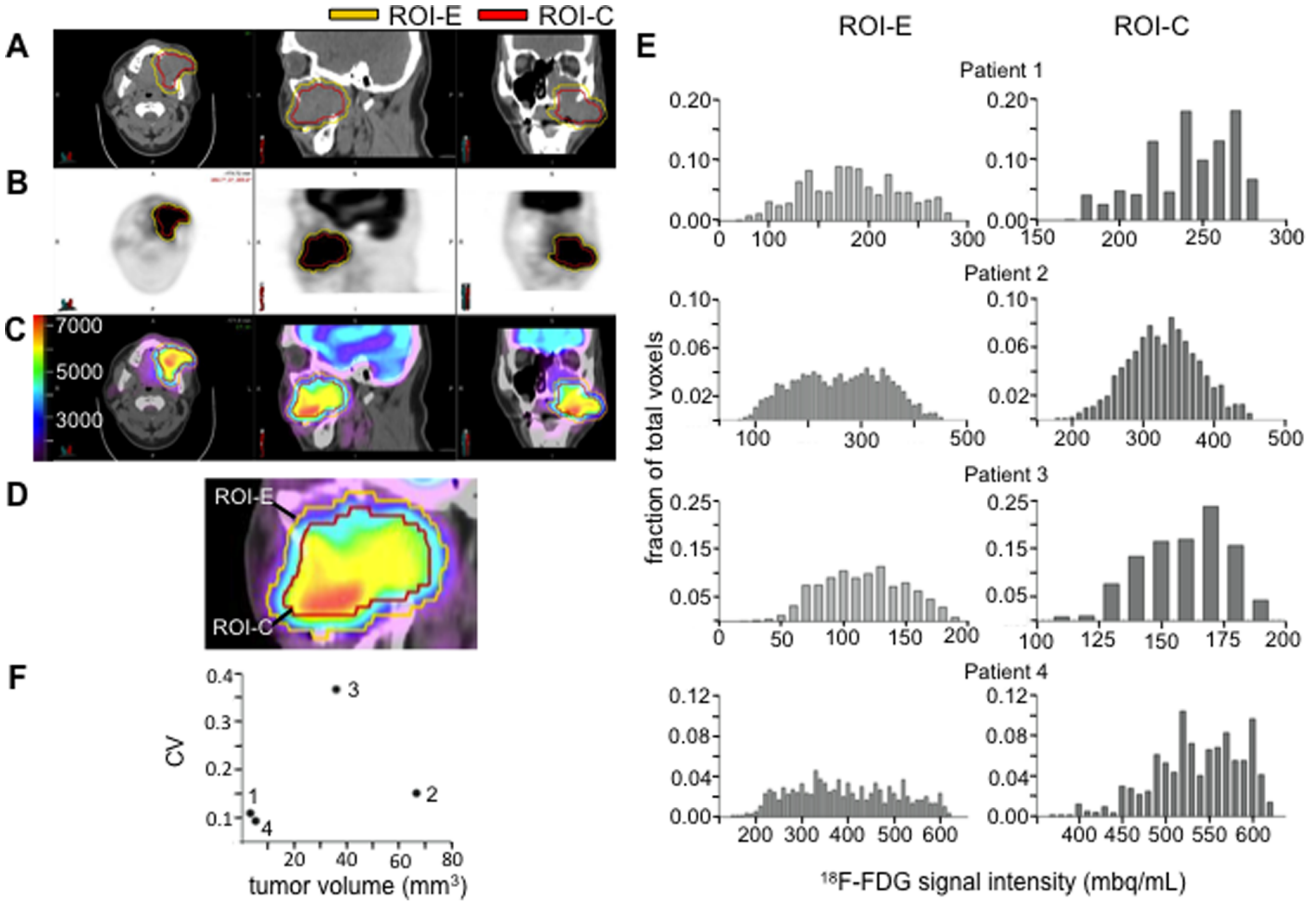
^18^F-FDG uptake in human HNSCC tumors is heterogeneous. A, Non-contrast head CT of patient 2. B, ^18^F-FDG signal on PET scan. C, Fused PET-CT image with scaled color bar (bq/mL). Yellow ROI represents ROI-E, established by PETEdge. Red ROI represent ROI-C, established by contracting ROI-E by 0.5 cm in all directions. D, Histograms of ^18^F-FDG signal in ROI-E or ROI-C intensity in each tumor. Binning was set at intervals of 10^3^ mbq/mL. Mean, maximum, minimum, and standard deviation of ^18^F-FDG signal within each tumor is shown in Table 2. E, Dot plot comparing tumor volume and coefficient of variance (CV) values established by ROI-C for each tumor. The numbers identify the same tumors numbered to the left of the histograms. Tumor volumes and CVs also listed in Table 2.

Having identified significant metabolic heterogeneity in a HNSCC xenograft model, we sought to measure metabolic heterogeneity in ^18^F-FDG PET scans of human HNSCC tumors by assessing CV values of tumor^18^F-FDG uptake. The PETEdge function of MIMVista software was used to generate a 3D ROI defining the tumor border (ROI-E). On several occasions, ROI-E encompassed the patient's airway or bone due to spillover of the ^18^F-FDG signal. Normal tissue surrounding the tumor had lower ^18^F-FDG signal relative to the tumor, and this difference between tumor and normal tissue would reduce the signal intensity of the adjacent tumor voxels due to partial volume effect [7], [9], [11], [13], [15], [33], [34]. To exclude non-tumor tissue, ROI-E was contracted circumferentially in 3D by 0.5 cm, the value of the PET scanner's resolution, creating ROI-C (Fig. 6A–C). The characteristics and ^18^F-FDG values recorded by PET-CT for each tumor are shown in Table 2. The intensity value of each voxel in ROI-E and ROI-C was plotted in a histogram to visualize the heterogeneity of ^18^F-FDG signal within the tumor. These histograms demonstrate that the transformation from ROI-E to ROI-C consistently removes the tumor regions with the lowest intensity of ^18^F-FDG signal that are barely above background.

**Table 2:** Tumor characteristics and ^18^F-FDG-PET values recorded from ROI-C using MIM software.

Patient	Tumor volume (mL)	Max (bq/mL)	Min (bq/mL)	Mean (bq/mL)	Median (bq/mL)	SD (bq/mL)	CV
1	3.19	287489.06	174075.95	246704.23	249721.62	27098.21	0.12
2	66.86	456195.81	155267.7	333665.19	333957.66	50224.01	0.15
3	36.15	261265.5	33070.44	148666.3	142562.28	52314.15	0.37
4	5.18	620795.06	371847.78	542301.38	544443.94	50281.7	0.09

SD, standard deviation. CV, coefficient of variance.

Similar to our assessment of 2-DG heterogeneity within the xenograft tumors, we calculated the CV value of ROI-C of each tumor. While two smallest tumors had the lowest CV values and the larger tumors had higher CV values, Figure 6F shows that tumor volume and CV are do not show a direct correlation. These data suggest that tumor metabolic heterogeneity may not be predictable based upon size alone, and that other factors, such as differential oxygenation and regional differences in cellular metabolomics could be driving tumor metabolic heterogeneity.

## Discussion

The purpose of this study was to quantify intratumoral metabolic heterogeneity visually by assessing the variability of 2-DG concentrations within HNSCC tumors. We began our investigation *in vitro* with two HNSCC cell lines that revealed distinct metabolic phenotypes despite being of similar malignant origin. OSC19 cells were highly glycolytic in normal culturing conditions, and hypoxic conditions (2% O_2_) stimulated even higher levels of glycolysis (Figs. 1 and 3F). OSC19 cells' increased reliance on glycolysis to generate ATP correlated with a lower level of OXPHOS activity at baseline, and an inability to modulate OXPHOS in response to hypoxia. Cal33 cells displayed higher OXPHOSat baseline, but when exposed to hypoxia, this cell line reduced its oxygen consumption in the first 16 hours of hypoxia exposure unlike OSC19 cells (Fig. 3A). After 48 hours of hypoxia exposure, Cal33 cells demonstrated further flexibility of their metabolic phenotype by increasing their glycolytic rate (Fig. 3F). While there was no significant difference in Cal33 OXPHOS activity after 48 hours of culturing in normoxic or hypoxic conditions, we believe this finding is a result of the normoxic cultures adopting slower metabolic activity and proliferation rate as the cellular density increased and nutrients were extracted from the media. Cells cultured in hypoxic conditions for 48 hours were forced to perform glycolysis at a faster rate in spite of increased cellular confluence and nutrient depletion due to the limited oxygen availability. These metabolic changes in Cal33 cells coincided with a rapid increase in HIF-1α stabilization during acute hypoxia (Fig. 2) and increased expression of PDHK1 and LDH-M after prolonged hypoxia exposure. This robust metabolic flexibility made Cal33 a promising cell line in which to investigate metabolic heterogeneity *in vivo*.

Data presented in Figure 4 indicated that we were able to measure for the first time molecular markers of metabolism and oxygenation simultaneously within live Cal33 xenograft tumors using high-resolution FMT imaging [14], [16], [18], [26], [35]. This method revealed regional differences of IR800-2-DG uptake within Cal33 tumors, indicating that portions of the tumors were highly glycolytic, and suggesting that other tumor regions were more reliant upon OXPHOS or other fuel sources. Tumor regions with detectable HypoxiSense accumulation had significantly higher IR800-2-DG signal as compared to the whole tumor, suggesting that the Cal33 cells adapted their metabolism in response to environmental pressures.

Most interestingly, tumors containing detectable HypoxiSense had greater intratumoral heterogeneity of IR800-2-DG signal than those tumors that lacked a hypoxic region as measured by their CV values (Fig. 4). This suggests that hypoxia promotes metabolic heterogeneity within tumors, and that measuring the heterogeneity of glucose uptake within a tumor may serve as a hypoxia marker. This relationship would prove especially valuable if it was present in human HNSCC tumors, as PET is limited to the detection of a single tracer at a time. Rajendran et al. [17], [31], [36] performed a pixel-by-pixel correlation of ^18^F-FDG and ^18^F-labeled fluoromisonidazole (FMISO), a marker of tissue hypoxia, in PET scans of 26 HNSCC tumors as well as several other tumor types. HNSCC tumors had the highest correlation of uptake between the two tracers of all the tumor types, however this relationship was weak. Tumor oxygenation can fluctuate rapidly, and marginal significance of the relationship between ^18^F-FDG and FMISO could be due to the fact that the PET scans were performed several days apart.

IHC of the xenograft tumors validated the *in vivo* association of greater metabolic heterogeneity in HypoxiSense positive tumors. While HIF-1α was detected in HypoxiSense negative and positive tumors, heterogeneity of HIF-1α, CAIX, and ATP5β expression was consistently greater in HypoxiSense positive tumors. The presence of HIF-1α and CAIX in HypoxiSense negative tumors suggests that HypoxiSense did not accumulate at a detectable level in these tumors despite the presence of hypoxia. CAIX expression at the cell surface in peri-necrotic tumor regions reflected the pattern of HIF-1α expression, a finding in agreement with previous studies [8], [12], [30], [32]. While CAIX expression alone has proven to be an unreliable independent marker of tumor hypoxia [7], [33], [34], [37], the combined expression of CAIX and HIF-1α was significantly predictive of a worsened prognosis for HNSCC patients [14], [16], [18], [30], [35], [38]. The variable expression of HIF-1α and CAIX within a tumor suggests that they may serve as valid biomarkers of intratumoral metabolic heterogeneity.

We extended our investigation of intratumoral heterogeneity to patient samples, and detected ^18^F-FDG-uptake heterogeneity in HNSCC PET scans of human patients (Fig. 6). Our method of contracting an algorithm-drawn ROI and eliminating 0.5 cm around the perimeter of each tumor aimed to reduce spurious ^18^F-FDG signal heterogeneity inherent at the tumor edge that arises from the mixing of lower ^18^F-FDG signal from normal tissues with higher^18^F-FDG signal from malignant tissue. We acknowledge that small portions of the tumor may have been excluded from our analysis, however we viewed this trade-off as necessary for a more accurate assessment of the overall tumor metabolic heterogeneity. The largest tumors in our analysis showed the greatest ^18^F-FDG uptake variability, however as previously noted in Figure 6 the largest tumor did not have the highest variability. A larger study will be necessary to better understand this relationship. Hatt et al [36] examined non-small cell lung cancer, and also concluded that the degree of metabolic heterogeneity was associated with larger tumor size.

While glucose can directly block cellular uptake of IR800-2-DG (data not shown, [30]), there is evidence that uptake and retention of IR800-2-DG and ^18^F-FDG may not directly coincide. Tseng et al reported that the uptake of ^18^F-FDG in gastrointestinal stromal xenograft tumors was reduced after treatment with nilotinib, and they regarded this phenomenon as a result of reduced metabolic activity within the treated tumors. Tumor uptake of IR800-2-DG, however, was not seen to decrease significantly after a three day treatment with nilotinib. The authors suggested that the structural differences in these tracers may alter their cellular import and/or retention [37]. IR800-2-DG remains widely used to assess tumor metabolism and follow tumor progression in several cell types [30], [38], and its intracellular accumulation has been correlated with GLUT-1 expression [30], [39]. While we acknowledge that there may be differences in the mechanisms of dye uptake and retention, our experiments clearly showed a marked heterogeneity in the uptake of this dye in xenograft tumors.

While tumor metabolism is routinely assessed in HNSCC tumors using ^18^F-FDG PET-CT scans, detecting glucose uptake in relation to hypoxia in HNSCC tumors has been inconclusive [23], [24], [40], [41]. Previous groups have investigated ^18^F-FDG intratumoral heterogeneity [40], [42]–[45], and our experiments, both *in vitro* and *in vivo*, support further investigation of intratumoral metabolic heterogeneity. We have established a platform for visualizing the consumption of other metabolites and assessing changes in metabolic heterogeneity when tumors are treated with metabolic inhibitors. Recognition of tumor metabolic heterogeneity may lead to improved therapy combinations and outcomes [2], [30]. In order to identify combination therapies with the greatest potential efficacy, the forces driving intratumoral heterogeneity must first be clarified. Differential tumor oxygenation and the variety of metabolites that a tumor can utilize influence tumor growth, and may contribute to the development of metabolically distinct populations. Our studies indicate that variable glucose uptake within a tumor provides information not only about the metabolic capabilities of the tumor, but may also serve as a marker of tumor hypoxia and the ability of tumor cells to respond to environmental pressures. Additionally, our work tested the feasibility of measuring ^18^F-FDG uptake variability in head and neck tumors on PET scans, which is currently the gold-standard for assessing tumor metabolism. ^18^F-FDG uptake variability in human tumors may be an indicator of tumor hypoxia, and prognosis, and therefore could guide a patient's treatment regimen. Validation in prospective clinical trials is warranted to translate these findings.

## Supporting Information

Figure S1 Seahorse assay records Cal33 and OSC19 glycolytic and oxidative activity.
(TIFF)

Figure S2 Hypoxia exposure does not alter CAIX expression significantly in Cal33 whole cell lysate.
(TIFF)

Figure S3 FMT detection of Hypoxisense, AngioSense, 2-DG in Cal33 xenograft tumors.
(TIFF)

Figure S4 Immunohistochemistry controls.
(TIFF)

Table S1 Fluorescent markers used to identify metabolic heterogeneity *in vivo*.
(TIFF)

Methods S1 Materials S1 describes the evaluation of cellular rates of glycolysis and oxidative phosphorylation using the Seahorse Flux Analyzer. Materials S2 describes the use of the fluorescent molecular tomography (FMT) and image analysis to visualize tumor metabolism *in vivo*.
(TIFF)
