# Supplementary figures and images for: Quantifying Metabolic Heterogeneity in Head and Neck Tumors in Real Time: 2-DG Uptake Is Highest in Hypoxic Tumor Regions
Source: PLoS One. 2014 Aug 15;9(8):e102452. doi: 10.1371/journal.pone.0102452 (PMC4134191; doi:10.1371/journal.pone.0102452)

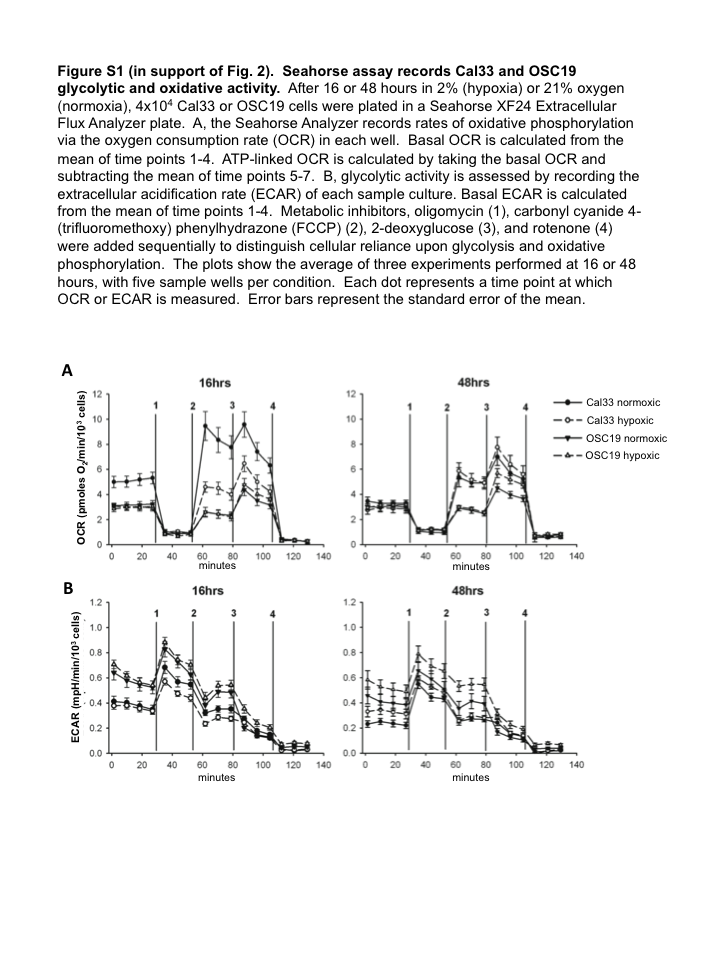

Supplement: Figure S1 — Seahorse assay records Cal33 and OSC19 glycolytic and oxidative activity. (TIFF) [file pone.0102452.s001.tiff]

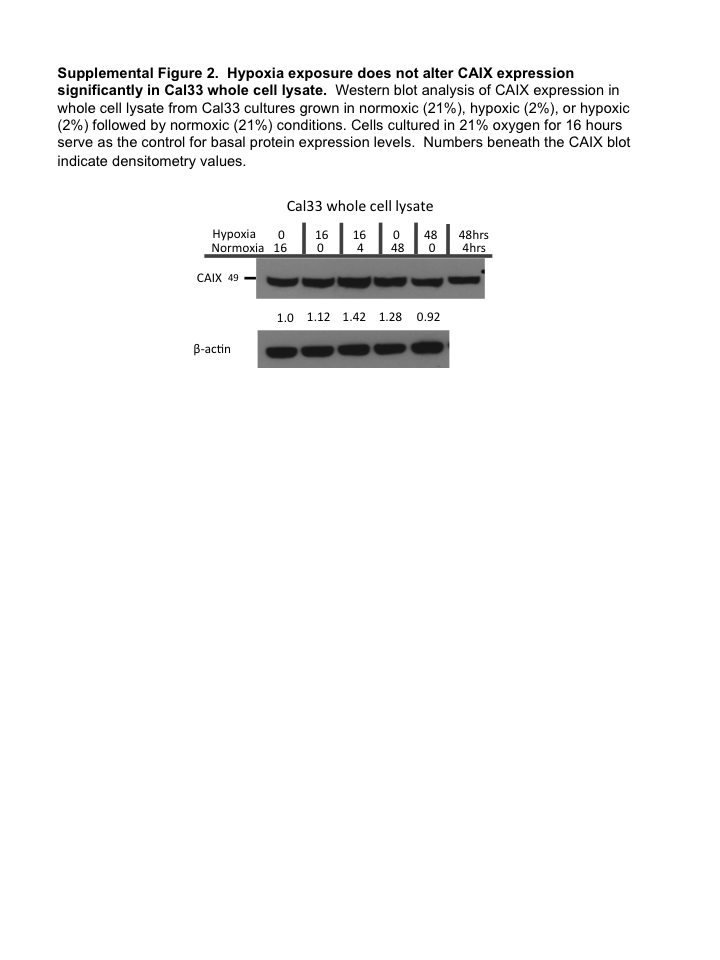

Supplement: Figure S2 — Hypoxia exposure does not alter CAIX expression significantly in Cal33 whole cell lysate. (TIFF) [file pone.0102452.s002.tiff]

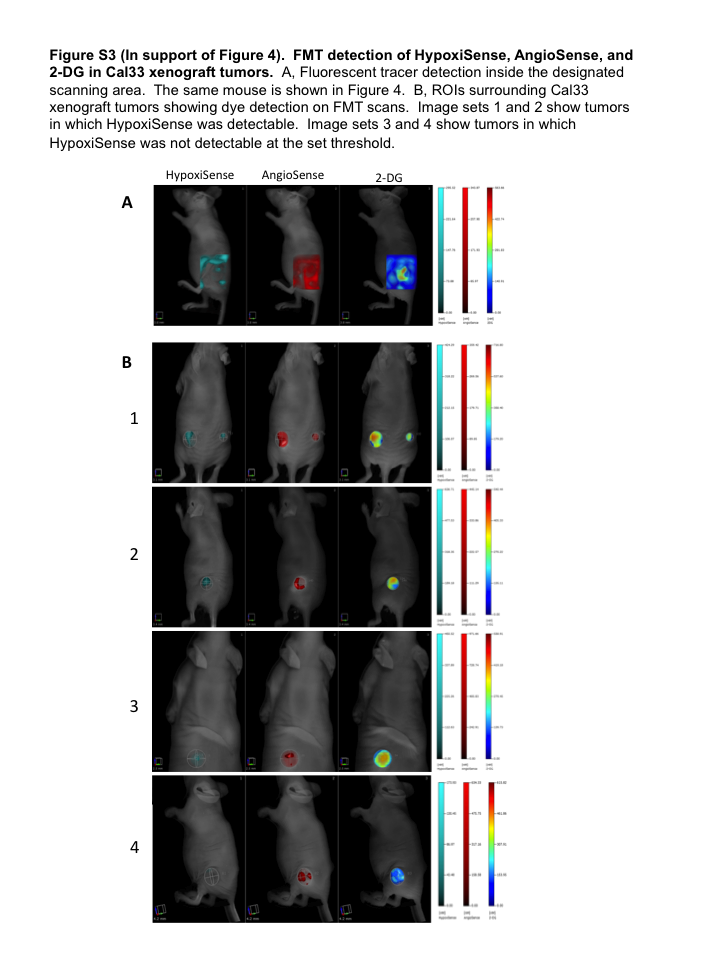

Supplement: Figure S3 — FMT detection of Hypoxisense, AngioSense, 2-DG in Cal33 xenograft tumors. (TIFF) [file pone.0102452.s003.tiff]

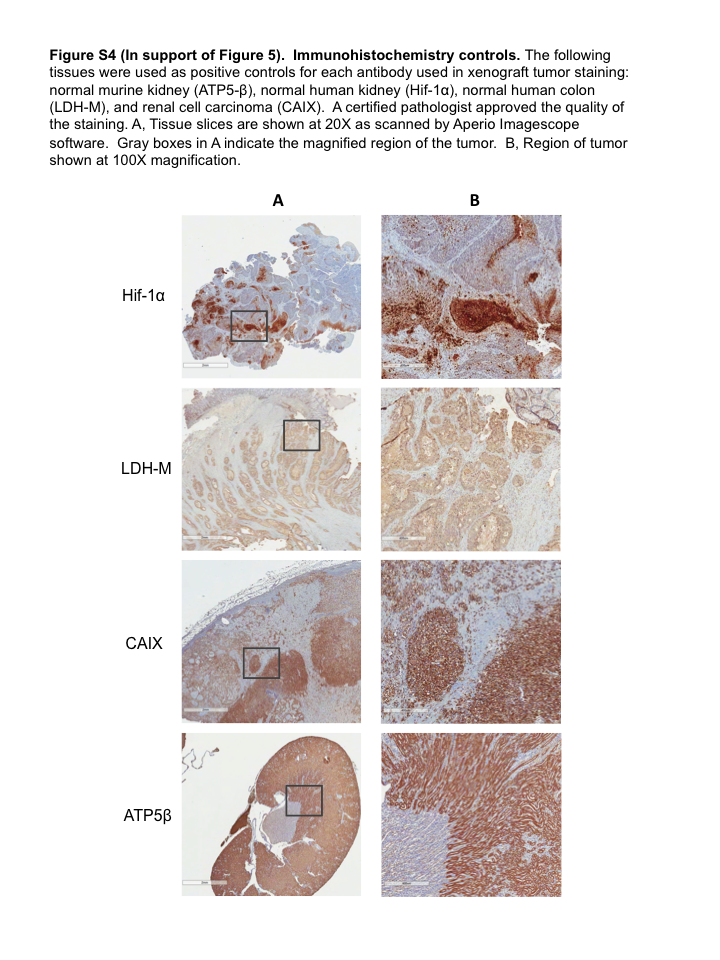

Supplement: Figure S4 — Immunohistochemistry controls. (TIFF) [file pone.0102452.s004.tiff]
